# Cyclin E/Cdk2-dependent phosphorylation of Mcl-1 determines its stability and cellular sensitivity to BH3 mimetics

**DOI:** 10.18632/oncotarget.4857

**Published:** 2015-07-15

**Authors:** Gaurav S. Choudhary, Trinh T. Tat, Saurav Misra, Brian T. Hill, Mitchell R. Smith, Alexandru Almasan, Suparna Mazumder

**Affiliations:** ^1^ Department of Cancer Biology, Lerner Research Institute, Cleveland Clinic, Cleveland, OH, USA; ^2^ Department of Molecular Cardiology, Lerner Research Institute, Cleveland Clinic, Cleveland, OH, USA; ^3^ Department of Immunology, Lerner Research Institute, Taussig Cancer Institute, Cleveland Clinic, Cleveland, OH, USA; ^4^ Department of Hematology and Oncology, Taussig Cancer Institute, Cleveland Clinic, Cleveland, OH, USA; ^5^ Department of Molecular Medicine, Cleveland Clinic Lerner College of Medicine of Case Western Reserve University, Cleveland, OH, USA; ^6^ Department of Pathology, Case Western Reserve University School of Medicine, Cleveland, OH, USA; ^7^ Department of Biochemistry, Case Western Reserve University School of Medicine, Cleveland, OH, USA

**Keywords:** Mcl-1, cyclin E, Cdk2, dinaciclib ABT-737, ABT-199, CLL

## Abstract

Cyclin E/Cdk2 kinase activity is frequently deregulated in human cancers, resulting in impaired apoptosis. Here, we show that cyclin E/Cdk2 phosphorylates and stabilizes the pro-survival Bcl-2 family protein Mcl-1, a key cell death resistance determinant to the small molecule Bcl-2 family inhibitors ABT-199 and ABT-737, mimetics of the Bcl-2 homology domain 3 (BH3). Cyclin E levels were elevated and there was increased association of cyclin E/Cdk2 with Mcl-1 in ABT-737-resistant compared to parental cells. Cyclin E depletion in various human tumor cell-lines and cyclin E^−/−^ mouse embryo fibroblasts showed decreased levels of Mcl-1 protein, with no change in Mcl-1 mRNA levels. In the absence of cyclin E, Mcl-1 ubiquitination was enhanced, leading to decreased protein stability. Studies with Mcl-1 phosphorylation mutants show that cyclin E/Cdk2-dependent phosphorylation of Mcl-1 residues on its PEST domain resulted in increased Mcl-1 stability (Thr92, and Thr163) and Bim binding (Ser64). Cyclin E knock-down restored ABT-737 sensitivity to acquired and inherently resistant Mcl-1-dependent tumor cells. CDK inhibition by dinaciclib resulted in Bim release from Mcl-1 in ABT-737-resistant cells. Dinaciclib in combination with ABT-737 and ABT-199 resulted in robust synergistic cell death in leukemic cells and primary chronic lymphocytic leukemia patient samples. Collectively, our findings identify a novel mechanism of cyclin E-mediated Mcl-1 regulation that provides a rationale for clinical use of Bcl-2 family and Cdk inhibitors for Mcl-1-dependent tumors.

## INTRODUCTION

The balance between cell proliferation and death is vital for normal growth, differentiation, and tissue homeostasis. Deregulation of proteins involved in these processes drive tumor progression and contributes to malignant phenotype. Tumorigenesis primarily involves overexpression of anti-apoptotic proteins Mcl-1, Bcl-2, and Bcl-xL [1] and deregulation of the cell cycle machinery due to increased cyclin E activity [2-4]. Cyclin E/cyclin-dependent kinase (Cdk) 2 plays a crucial role in signaling cascades that govern phosphorylation of a wide range of substrates. These include E2F, Rb, nucleophosmin, and NPAT (nuclear protein, ataxia-telangiectasia), which contain a Cdk2-binding motif and are involved in both physiological and pathological cellular processes [3, 5]. Oncogenic signaling by cyclin E gives rise to malignant phenotypes characterized by genomic instability, impaired apoptosis, and drug resistance, as observed in breast, ovarian, lung, and hematologic malignancies [5].

Mcl-1 is a labile pro-survival Bcl-2 family member that has variable expression levels during the cell cycle, suggesting that cell cycle regulatory proteins modulate Mcl-1 protein levels [6, 7]. Mcl-1 plays a prominent role in evading mitochondrial outer membrane permeabilization by sequestering Bcl-2-homology domain 3 (BH3)-only proteins in response to various apoptotic stimuli. Mcl-1 is also involved in critical physiological processes, such as mitochondrial homeostasis, apoptosis, autophagy and cell cycle regulation, as well as maintenance and development of hematopoetic stem cells [8]. Post-translational modifications of Mcl-1, primarily by phosphorylation, have a significant contribution to the control of Mcl-1 expression that determines cellular state as well as cellular fate. The N-terminal proline, glutamic acid, serine and threonine regions (PEST) domain of Mcl-1 encompass 170 residues that are unique among Bcl-2 family members. They contain putative regulatory residues and motifs responsible for Mcl-1 phosphorylation, proteolytic cleavage, and ubiquitination [1, 8]. Phosphorylation of Mcl-1 at Thr92, Ser121, and Thr163 residues, which is primarily mediated by ERK-1/JNK/p38, results in Mcl-1 stabilization [9, 10]. Glycogen synthase kinase-3β (GSK-3β) destabilizes Mcl-1 by phosphorylating Ser155 and Ser159 [11, 12]. However, this process requires a priming kinase, ERK/JNK, to phosphorylate Mcl-1 Thr163 [8]. Cell-cycle-associated phosphorylation of Mcl-1 by Cdk1/Cdk2 facilitates Bim sequestration [7] or degradation during mitosis [6, 13]. Although regulation of Mcl-1 during mitosis has been reported [14], the role of enhanced cyclin E/Cdk2 activity in impaired apoptosis and drug resistance has not been defined.

ABT-737/ABT-263 (navitoclax) and ABT-199 (venetoclax) are small molecules that inhibit Bcl-2 family proteins by binding to two hydrophobic pockets formed by the BH3 domain contained in all Bcl-2 family members [15]. ABT-199 has shown dramatic clinical activity in Bcl-2-dependent hematologic malignancies, most notably in chronic lymphocytic leukemia (CLL) and mantle cell lymphoma [16]. We have previously reported that Mcl-1 levels determine sensitivity to both ABT-737 and ABT-199 in acquired and inherently resistant leukemic cells [17, 18]. Mcl-1 anti-apoptotic function in ABT-737-resistant (ABT-R) cells is enhanced by phosphorylation on its Thr163 and Ser64 residues, which increases Mcl-1 stability and Bim sequestration, respectively [17]. In this study, we show that both cyclin E and Mcl-1 expression are increased in ABT-R cells. We define Mcl-1 as a substrate of cyclin E/Cdk2 that is phosphorylated at Ser64, Thr92, and Thr163, modifications that are critical for Mcl-1 stability and function. Targeting cyclin E using Cdk inhibition, alone or in combination with the BH3 mimetics ABT-737 or ABT-199 substantially reduces survival of Mcl-1-dependent tumor cells. Our data reveal a novel functional role of cyclin E/Cdk2 in Mcl-1 regulation and provide strategies to overcome Mcl-1-mediated drug resistance in lymphoid malignancies as well as solid tumors.

## RESULTS

### Cyclin E regulates Mcl-1 protein levels

**Figure 1:**
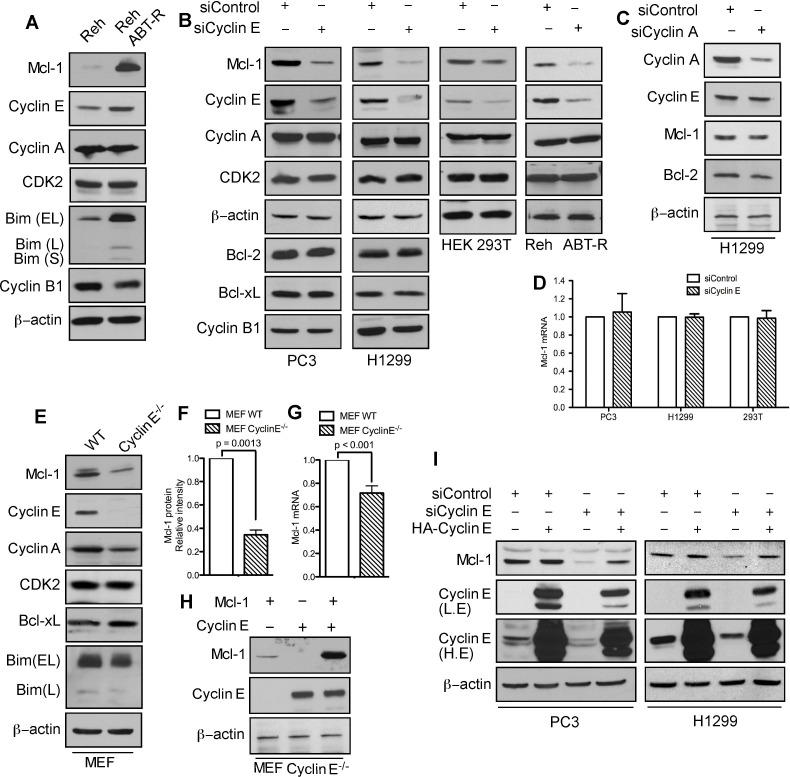
Mcl-1 protein levels are regulated by cyclin E/Cdk2 **A.** Immunoblot analysis for the indicated proteins in Reh parental and ABT-R-derivative cells. Cells were transfected with *siControl* and **B.** siCyclin E or **C.** siCyclin A and expression levels of the indicated proteins was determined by immunoblotting. **D.** *Mcl-1* mRNA was analyzed by qRT-PCR in cells transfected with siControl or siCyclin E. WT and cyclin E^−/−^ MEFs were analyzed for: **E.** Expression levels of the indicated proteins by immunoblotting and **F.** Expression levels of Mcl-1 that were quantified by ImageJ. **G.** *Mcl-1* mRNA fold change was determined by qRT-PCR. **H.** Expression levels of Mcl-1 and cyclin E in cyclin E^−/−^ MEFs after expression of Mcl-1 and Cyclin E constructs, alone or together. **I.** PC3 and H1299 cells were transfected with siControl and siCyclin E. After 24 h, these cells were transfected with HA-cyclin E and expression levels of Mcl-1 and cyclin E were determined by immunoblotting. The data in **A.**-**C.**, **E.**, **H.**-**I.** are representative of three independent experiments. β-actin was used as a loading control. mRNA levels of control cells were set to 1. *P* values in **F.**-**G.** were obtained by a two-tailed Student's *t*-test. Bar graphs are represented as mean ± SD (*n* = 3).

In cells that developed resistance to the Bcl-2 inhibitor ABT-737, phosphorylation of Mcl-1 on Thr163 and Ser64 increased its stability and Bim sequestration [17]. Immunoblot analyses indicate that in addition to Mcl-1, cyclin E levels were also higher in ABT-R compared to parental cells. There was no change in Cdk2 and cyclin A, while cyclin B1 levels were decreased (Figure 1A). To investigate how cyclin E affects Mcl-1 expression, cyclin E levels were down-regulated in PC3, H1299, HEK293T, and Reh ABT-R cells by siRNA-mediated knockdown. Mcl-1 levels were diminished significantly upon knockdown of cyclin E, but not cyclin A. Cyclin E knockdown had no effect on the levels of Cdk2, cyclin A, cyclin B1, and pro-survival Bcl-2 proteins (Figure 1B and 1C). In siCyclin E-expressing cells there was no change in *Mcl-1* mRNA levels (Figure 1D). Moreover, no significant differences in the cell cycle profile were observed in ABT-R compared to parental cells or after cyclin E knockdown in PC3 and H1299 cells (Supplementary Figure S1A and S1B). To conclusively demonstrate that cyclin E regulates Mcl-1 expression, we investigated Mcl-1 levels in cyclin E^−/−^ mouse embryonic fibroblasts (MEFs) [19]. There was a two-fold decrease in Mcl-1 protein expression in cyclin E^−/−^ compared to wild type (WT) MEFs, with minimal reduction in *Mcl-1* mRNA levels (Figure 1E, 1F, and 1G). Reconstitution of cyclin E expression in cyclin E^−/−^ MEFs with HA-cyclin E confirmed that Myc-Mcl-1 levels were higher in the presence of constitutively active cyclin E (Figure 1H). Importantly, HA-cyclin E also rescued endogenous Mcl-1 levels that were initially decreased after downregulating cyclin E (Figure 1I). These results suggest that cyclin E regulates Mcl-1 protein predominantly at post-translational level.

### Cyclin E/Cdk2 associates with and stabilizes Mcl-1 protein

**Figure 2:**
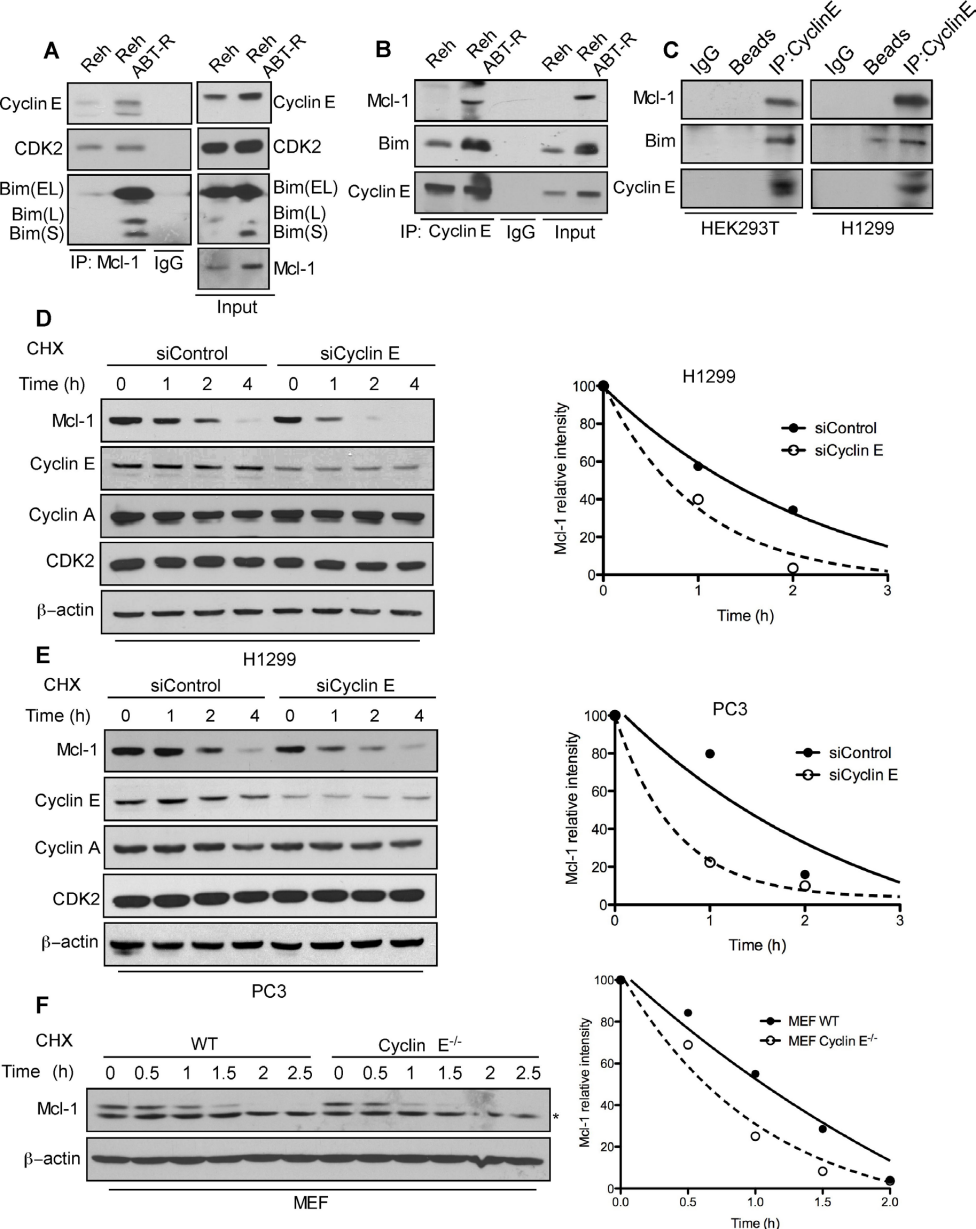
Association with the cyclin E/Cdk2 complex regulates Mcl-1 stability Association of Mcl-1 with cyclin E and Cdk2 was determined by immunoblotting in Reh and Reh ABT-R cells following immunoprecipitation of: **A.** Mcl-1 and **B.** cyclin E **C.** Association of cyclin E with Mcl-1 and Bim was determined in HEK293T and H1299 cells. Cyclin E levels were downregulated in H1299 and PC3 cells by siCyclin E. Mcl-1 protein half-life was determined by treating: **D.** H1299, **E.** PC3, and **F.** WT and cyclin E^−/−^ MEFs with cycloheximide (20 μg/ml) for the indicated time followed by immunoblotting. β-actin was used as loading control. Data in **D.**-**F.** were quantified by ImageJ. Data in **A.**-**F.** are representative of three independent experiments.

To define the mechanism responsible for cyclin E-mediated Mcl-1 regulation, we examined the interaction of Mcl-1 with cyclin E and its consequence on Mcl-1 stability. Mcl-1 was immunoprecipitated from parental and ABT-R Reh cells. Immunoblotting analyses indicate an increased association of cyclin E and Bim with Mcl-1 in ABT-R compared to parental cells (Figure 2A). This association was confirmed by reciprocal immunoprecipitation with cyclin E and immunoblotting for Mcl-1 (Figure 2B). There was no association of cyclin E with the pro-survival protein Bcl-2 in Reh parental and ABT-R cells (Supplementary Figure S2). Similar interactions between Mcl-1 and Cyclin E were detected in HEK293T and H1299 epithelial tumor cells (Figure 2C).

Mcl-1 is a labile protein that has a short half-life [1, 8]. In order to understand the functional importance of its association with cyclin E/Cdk2, we determined the half-life of Mcl-1 in cyclin E depleted cells in presence of cycloheximide, a protein synthesis inhibitor. Mcl-1 half-life was substantially decreased in siCyclin E-expressing H1299, PC3 and in cyclin E^−/−^ MEFs (Figure 2D-2F). These findings indicate that the cyclin E/Cdk2 complex increases Mcl-1 stability, as indicated by its decreased protein turnover.

### Cyclin E/Cdk2 mediates Mcl-1 phosphorylation and Bim sequestration

**Figure 3:**
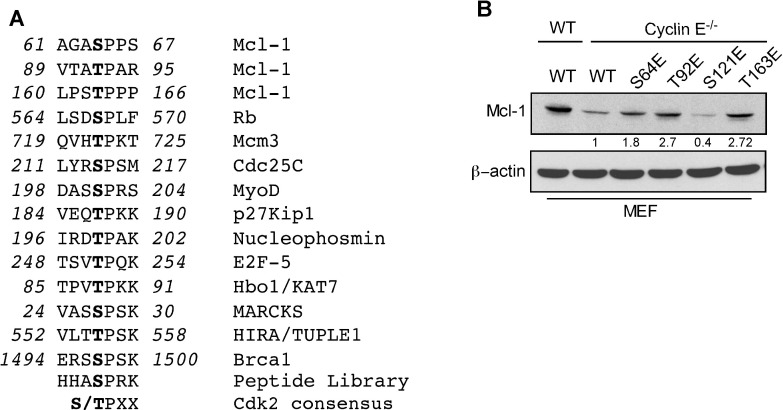
Identification of Mcl-1 phosphorylation sites by Cyclin E/Cdk2 **A.** Sequence alignment of the optimum amino-acid sequence predicted for Cdk2 with Mcl-1 and known Cdk2 phosphorylation substrate motifs. **B.** Immunoblot analysis for Mcl-1 in WT and cyclin E^−/−^ MEFs after expressing Myc-Mcl-1 WT, S64E, T92E, S121E, and T163E constructs. β-actin was used as a loading control. Data in **B.** was quantified by ImageJ. These data are representative of three independent experiments.

The Mcl-1 N-terminal domain contains putative regulatory motifs that are rich in PEST residues, and is predicted to have a low structural complexity [8]. To determine how cyclin E/Cdk2 phosphorylated Mcl-1 to enhance its stability and function, we defined the sequence S/TPXX as the Mcl-1 motif that is phosphorylated by cyclin E/Cdk2 (Figure 3A and Supplementary Figure S3A). This sequence is similar to X_-1_(S/T_0_)P_+1_X_+2_(K/R+3) that is present in known Cdk2 substrates [20]. Substrate affinity studies show that short synthetic peptides containing such sequences act as efficient substrates for cyclin-Cdk complexes [20]. Structural modeling of selected S/TPXX sequences within the Mcl-1 PEST domain supports the notion that these sites can be phosphorylated by cyclin E/Cdk2 (Supplementary Figure S3B-S3I).

**Figure 4:**
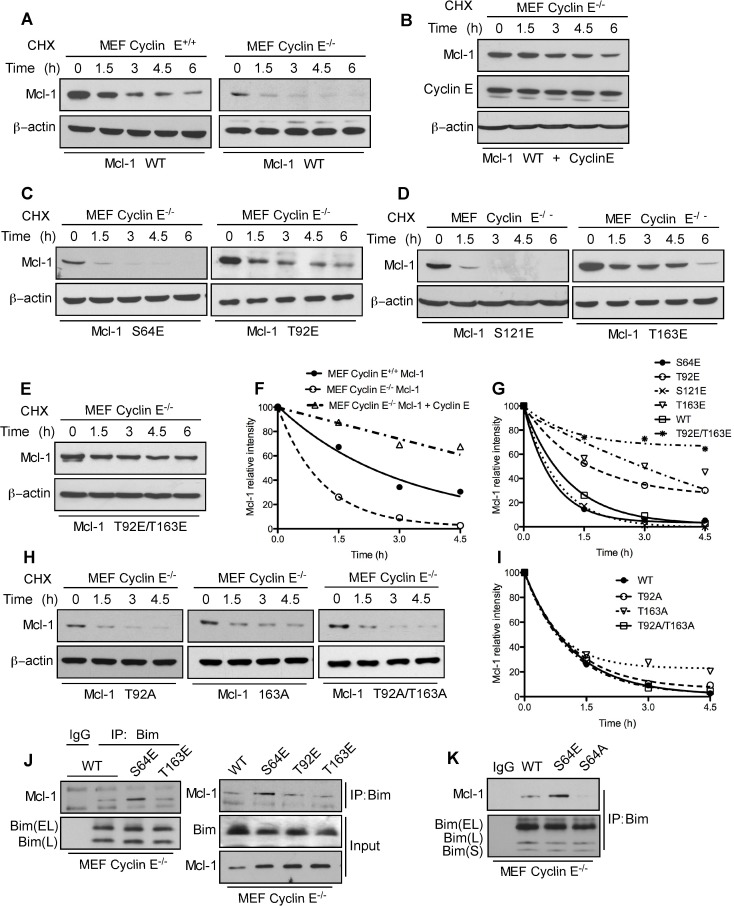
Mcl-1 stability and Bim sequestration is dependent on cyclin E/Cdk2 Mcl-1 protein half-life was determined by expressing WT Myc-Mcl-1 **A.** individually in cyclin E^+/+^ and cyclin E^−/−^ MEFs **B.** together with HA-cyclin E in cyclin E^−/−^ MEFs and then treating with cycloheximide for the indicated time, followed by immunoblotting. Immunoblot analysis of cyclin E^−/−^ MEFs transfected with **C.** S64E, T92E, **D.** S121E, T163E **E.** T92E/T163E **H.** T92A, T163A and T92A/T163A Mcl-1 mutants and treated with cycloheximide for the indicated time. Data in **F.**, **G.**, **I.** were quantified by ImageJ. Cyclin E^−/−^ MEFs were transfected with **J.** Myc-Mcl-1 WT, S64E, T92E and T163E **K.** Myc-Mcl-1 WT, S64E and S64A. After 24 h, Bim was immunoprecipitated and its association with Mcl-1 was analyzed by immunoblotting. β-actin was used as loading control. These data are representative of three independent experiments.

To directly test the role of cyclin E/Cdk2-dependent phosphorylation of Mcl-1, we generated phosphomimetic and alanine mutants at sites within the PEST domain (Ser64, Thr92, Ser121, and Thr163) that matched the putative Cdk2 phosphorylation motif. Myc-tagged Mcl-1-S64E, T92E, and T163E proteins exhibited higher steady-state levels than WT-Mcl-1 when expressed in cyclin E^−/−^ MEFs, while the Mcl-1 S121E expression level was lower (Figure 3B). These results indicate that the ^64^SPPS^67^, ^92^TPAR^95^, and ^163^TPPP^166^ Mcl-1 motifs are substrates for cyclin E/Cdk2-dependent phosphorylation. There was no significant change in expression levels of Mcl-1 phosphomimetic mutants in WT MEFs (Supplementary Figure S4A) with the exception of Mcl-1 T92E/T163E. Thr92 and Thr163 phosphorylation enhance Mcl-1 stability [8]; hence the observed elevated Mcl-1 T92E/T163E levels were expected. There was no change in expression levels of Mcl-1 alanine mutants in WT and cyclin E^−/−^ MEFs (Supplementary Figure S4B-S4C). In order to understand the direct impact of cyclin E/Cdk2-mediated phosphorylation on Mcl-1 stability, we expressed Myc-Mcl-1, alone and together with HA-cyclin E, in WT and cyclin E^−/−^ MEFs. Importantly, the Mcl-1 half-life was substantially decreased in cyclin E^−/−^ compared to WT MEFs and then increased substantially when HA-cyclin E was co-expressed (Figure 4A, 4B, and 4E). Subsequent half-life studies with Myc-Mcl-1 mutants revealed that phosphorylation at Thr92 and Thr163, but not Ser64 or Ser121, increased Mcl-1 level and half-life as compared to the WT-Mcl-1 construct in cyclin E^−/−^ MEFs (Figure 4C-4G). Mcl-1 half-life with Myc-Mcl-1 T92A, T163A and T92A/T163A was comparable with WT-Mcl-1 in cyclin E^−/−^ MEFs (Figure 4H, 4I). These data suggest that cyclin E/Cdk2 stabilizes Mcl-1 by phosphorylating Thr92 and Thr163.

We reported that ABT-R cells have increased Mcl-1:Bim interaction when Mcl-1 is phosphorylated on Ser64 [17]. To conclusively determine whether the cyclin E/Cdk2-dependent phosphorylation sites on Mcl-1 affect its interaction with Bim, endogenous Bim was immunoprecipitated from cyclin E^−/−^ MEFs transfected with Myc-tagged WT or the S4E T92E, T163E or S64A Mcl-1 mutants. Immunoblot analyses indicated an increased association of Bim with S64E Mcl-1 as compared to WT, T92E and T163E Mcl-1 (Figure 4J). Interestingly, Mcl-1:Bim association was completely abolished when Mcl-1-S64A construct was used as compared to Mcl-1 WT and S64E (Figure 4K). This finding suggests that cyclin E/Cdk2-mediated phosphorylation on Ser64 enhances the anti-apoptotic function of Mcl-1 by Bim sequestration. These results indicate that Mcl-1 phosphorylation, mediated by cyclin E/Cdk2, enhances Mcl-1 stability and function.

### Mcl-1 ubiquitination is regulated by cyclin E/CDK2

**Figure 5:**
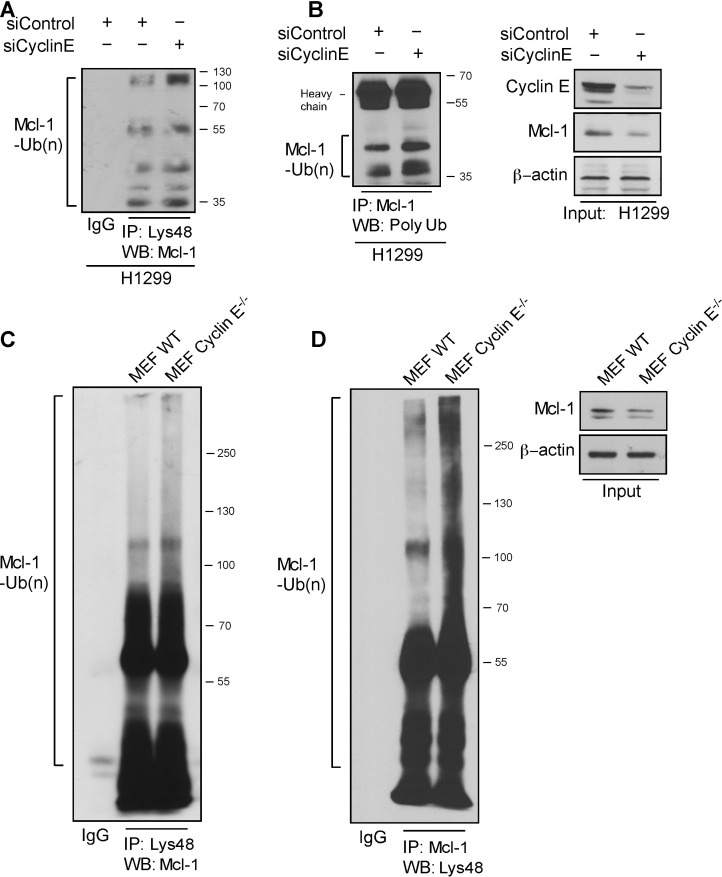
Cyclin E regulates Mcl-1 ubiquitination Ubiquitination of Mcl-1 was determined in H1299 cells transfected with siCyclin E and siControl by immunoprecipitating with: **A.** Lys48-specific antibody and immunoblotting with Mcl-1 and **B.** Mcl-1-specific antibody and immunoblotting for polyubiquitinated proteins. Input is shown in the left panel. **C.** Lys48-linked proteins were immunoprecipitated in WT and cyclin E^−/−^ MEFs and Mcl-1 ubiquitination was determined by a Mcl-1-specific antibody. **D.** Reciprocal immunoprecipitation for Mcl-1 was performed in WT and cyclin E^−/−^ MEFs and Mcl-1 polyubiquitination was determined by immunoblotting (left panel). Mcl-1 expression levels (right panel). Data from **A.**-**D.** are representative of three independent experiments.

Given the increased turnover of Mcl-1 in ABT-R cells, we investigated whether association with cyclin E/Cdk2 affects its ubiquitination. Immunoprecipitation of Lys48-linked proteins from siCyclin E-expressing H1299 cells and immunoblotting for Mcl-1 revealed increased association of Lys48-linked ubiquitin chains (Figure 5A). Additionally, silencing of cyclin E resulted in an increase in total poly-ubiqutinated Mcl-1 (Figure 5B). Similarly, Mcl-1 ubiquitination was enhanced in Lys48-linked poly-ubiquitin immunoprecipitates from cyclin E^−/−^ compared to WT MEFs (Figure 5C). Reciprocal immunoprecipitation with Mcl-1 and immunoblotting for Lys48-linked poly-ubiquitin confirmed these observations (Figure 5D). These results indicate that diminishing cyclin E expression either by knockdown or knockout approaches enhances poly-ubiquitination and ubiquitin-mediated degradation of Mcl-1.

### BH3 mimetics in combination with Cdk inhibitors overcome cyclin E/Cdk2-mediated chemoresistance

**Figure 6:**
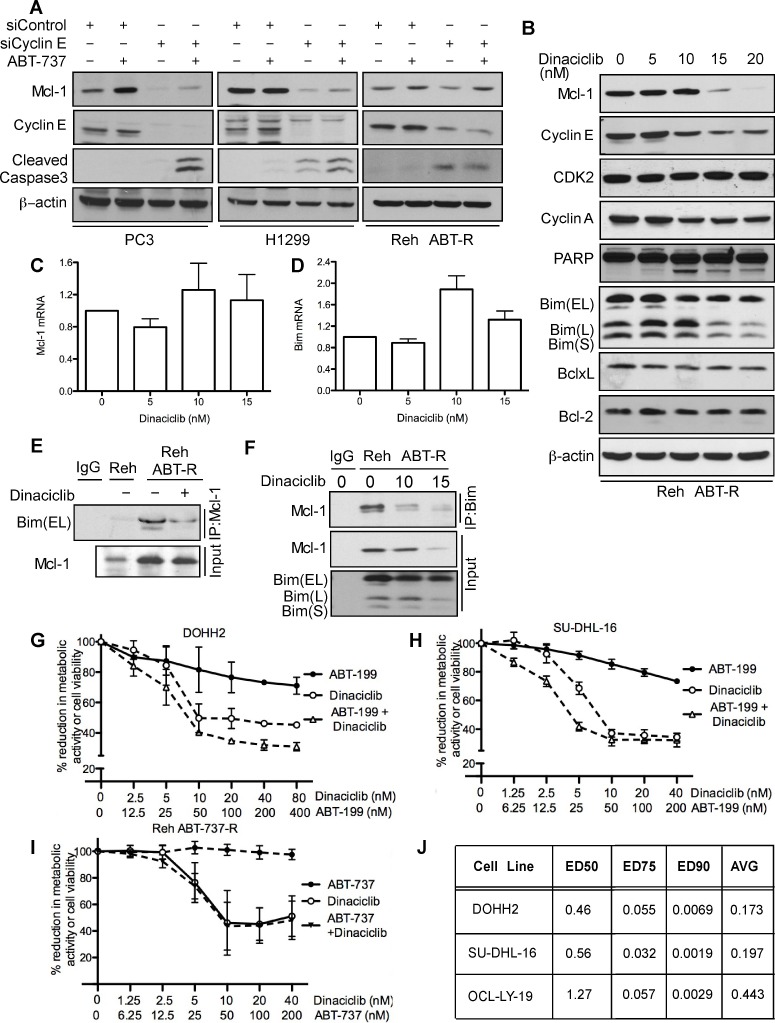
Cyclin E/Cdk2 inhibition sensitizes tumor cells to ABT-737/ABT-199 **A.** PC3, H1299, and Reh ABT-R cells were transfected with siCyclin E and siControl and treated with ABT-737 (10 μM) after 24 h. Expression levels of the indicated proteins were determined by immunoblotting after an additional 18 h of incubation. Reh ABT-R cells were treated with dinaciclib for 24 h and **B.** expression levels for the indicated proteins was determined by immunoblotting **C.** Mcl-1 and **D.** Bim mRNA was analyzed by qRT-PCR. **E.** Reh ABT-R cells were treated with dinaciclib (10 nM) for 18 h. Association of Mcl-1 with Bim was determined by immunoblotting in Reh and Reh ABT-R cells by immunoprecipitating Mcl-1. **F.** Association of Bim with Mcl-1 was determined by immunoprecipitating Bim in Reh ABT-R cells after treating with dinaciclib for 18 h. **G.** DOHH2 and **H.** SU-DHL-16 **I.** Reh ABT-R cells were treated with the indicated concentrations of ABT-199 ± dinaciclib for 24 h. Percentage reduction in metabolic activity was determined by the MTS assay. **J.** Combination index (CI) values for indicated cell lines. CI< 1 indicates synergism. Data in **A.**, **B.**, **E.**, **F.** are representative of three independent experiments. SD in **C.**, **D.**, **G.**-**I.** is indicated by error bars (*n* = 3).

As many tumors depend on Mcl-1 for chemoresistance [1, 21, 22], we investigated whether targeting cyclin E/Cdk2 could sensitize cancer cells to BH3 mimetics. Mcl-1 levels were decreased in PC3 and H1299 cells following cyclin E knockdown. Caspase-3 was proteolytically cleaved following ABT-737 treatment only in PC3 and H1299 cells with reduced cyclin E levels thus overcoming Mcl-1 mediated ABT-737 resistance (Figure 6A). In Reh ABT-R cells Mcl-1 levels were down-regulated after cyclin E depletion and caspase-3 was cleaved. However, there was no additional increase in caspase-3 cleavage with ABT-737 treatment (Figure 6A). Further extending this approach to targeting Cdks pharmacologically with the small-molecule inhibitor dinaciclib (SCH727965; 10 nM) resulted in reduced cyclin E and cyclin A levels, PARP cleavage, and decrease in metabolic activity (Figure 6B and 6H). No changes were seen in mRNA levels of Mcl-1 and Bim (Figure 6C and 6D). There was no change in expression levels of Mcl-1 and other Bcl-2 family proteins at lower concentrations of dinaciclib. To study the effect of dinaciclib on Mcl-1:Bim interaction, Mcl-1 was immunoprecipitated in parental and resistant Reh cells following dinaciclib treatment. Immunoblotting analyses with Bim indicate that there was higher Mcl-1:Bim association in Reh ABT-R compared to parental cells that was disrupted following dinaciclib treatment (Figure 6E). This result was confirmed by reciprocal immunoprecipitation with Bim and immunoblotting for Mcl-1 after dinaciclib treatment (Figure 6F).

**Figure 7:**
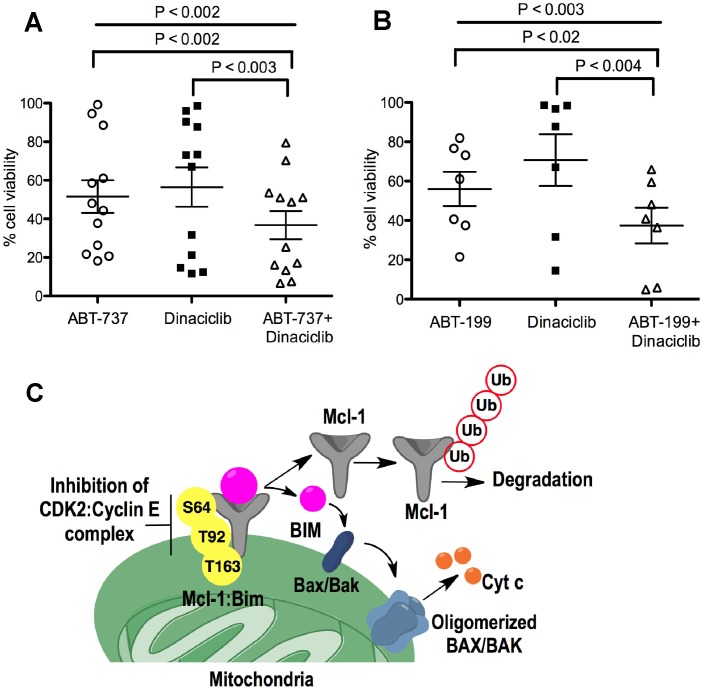
Dinaciclib sensitizes CLL cells to ABT-737 and ABT-199 CLL cells were treated for 24 h with: **A.** ABT-737 (10 nM), **B.** ABT-199 (10 nM) alone or in combination with dinaciclib (10 nM). Cell viability was determined by Annexin V-PI staining, represented as percentage relative to control cells treated with DMSO. *P* values were obtained by two-tailed Student's *t*-test and one-way ANOVA. **C.** Schematic illustration of the role of Cyclin E/Cdk2 in Mcl-1 protein regulation. Inhibition of cyclin E/Cdk2 destabilizes Mcl-1 by inhibiting its phosphorylation on Ser64, Thr92, and Thr163 residues resulting in: (i) Mcl-1 ubiquitination and degradation and (ii) Bim release from the Mcl-1-Bim complex, causing Bax activation and cell death.

Diffuse Large B-Cell Lymphoma (DLBCL) DOHH2 and SU-DHL-16 cells, which have high Mcl-1 protein levels and are resistant to ABT-199 [18], showed high sensitivity to dinaciclib + ABT-199 (Figure 6G, 6H), a combination that was highly synergistic (CI<0.2) (Figure 6J). DLBCL OCL-LY-19 cells with low Mcl-1 levels that are sensitive to ABT-199 [18] showed similar synergism to the combination of these two agents (Figure 6J). There was no additional cell death with the dinaciclib + ABT-737 combination as compared with dinaciclib alone treatment in Reh ABT-R (Figure 6I). Importantly, primary patient-derived CLL cells showed high sensitivity to dinaciclib + ABT-737 or ABT-199, as indicated by cell viability analyses with Annexin V-PI and flow cytometry (Figure 7A and 7B). These findings indicate that inhibiting cyclin E/Cdk2 activity enhances the sensitivity of Mcl-1-dependent tumors to BH3 mimetics.

## DISCUSSION

Mcl-1 plays a pivotal role in evading cell death in response to various apoptotic stimuli in solid tumors and hematologic malignancies [8]. Cancer cells modulate Mcl-1 by exploiting a number of redundant pathways regulating at transcriptional, translational, and post-translational levels. Regulation of Mcl-1 at post-translational level is primarily mediated by phosphorylation by Cdk1, Cdk2, JNK, ERK, and GSK-3β [1]. Upstream signaling pathways regulate these kinases that phosphorylate the Mcl-1 PEST domain, which plays a decisive role in Mcl-1 stability [23, 24] and binding of BH3-only proteins [7]. Based on the experimental evidence presented here and our previous findings [17], we propose a mechanism for cyclin E/Cdk2-dependent Mcl-1 regulation in mediating chemoresistance to Bcl-2 inhibitors. We define cyclin E, but not cyclin A, as a regulatory partner for Cdk2 activity that stabilizes Mcl-1 and determines the response to BH3 mimetics (Figure 7C). We found that in the absence of cyclin E in ABT-737 resistant cells, Mcl-1 protein levels were substantially reduced, with no effect on *Mcl-1* mRNA. Previous studies have shown that Mcl-1 levels are a critical resistance determinant to ABT-737 and ABT-199 treatment [17, 18, 25]. Mcl-1 stability, that was enhanced in acquired and inherent ABT-737/199 resistant, as compared to sensitive cells [17, 18], was prevented in the absence of cyclin E. Post-translational modification on Mcl-1 N-terminal region determine its stability [26]. We show that the Mcl-1 N-terminal region has Cdk2-specific phosphorylation motifs at Ser64, Ser121, Thr92, and Thr163 residues. Except Ser121, levels of mutant Mcl-1 with phosphomimetic changes at Ser64, Thr92, and Thr163 were higher than WT Mcl-1. As expected, conversion of Thr92, Thr163, and Ser64 to alanine decreased Mcl-1 stability and ability to sequester Bim. Consistent with our results, it has been reported that Ser121 phosphorylation did not have any effect on Mcl-1 stability [27] and hence did not increase its protein levels. We show that cyclin E/Cdk2-mediated phosphorylation at: (i) Thr92 and Thr163 increased Mcl-1 stability and (ii) Ser64 increased Mcl-1's ability to sequester Bim. Our results regarding the function of these residues on Mcl-1 are consistent with previous reports [7, 8, 23, 24]. Mcl-1 levels predict the response to anti-tubulin chemotherapeutics and ABT-737 in patients harboring loss-of-function in the *FBW7* (F-box and WD repeat domain containing 7) tumor suppressor gene [13, 28, 29]. Interestingly, cyclin E is also a target of FBW7 [30] and *FBW7* mutations deregulate cyclin E and Mcl-1 levels in primary tumors [31]. We show that Mcl-1 phosphorylation by cyclin E/Cdk2 is critical for diminished Mcl-1 ubiquitination, thereby preventing cell death.

Targeting Mcl-1 by induction of the BH3-only protein Noxa [17] or by inhibiting MEK [32] or PI3K [18, 33] pathway can accelerate ABT-737- and ABT-199-mediated cell death. As these Bcl-2 inhibitors do not target Mcl-1, it limits their applicability to solid tumors where Mcl-1 is abundant [21, 22]. Based on our findings, Mcl-1 protein levels were decreased following cyclin E depletion. ABT-737 treatment in solid tumors depleted of cyclin E resulted in proteolytic clevage of caspase-3. However, in acquired ABT-R cells depleted of cyclin E there was no further activation of caspase-3 following ABT-737 treatment. This is expected, as ABT-R cells are Mcl-1 dependent and therefore Bim is sequestered by Mcl-1 and not Bcl-2 or Bcl-xL [17]. Since ABT-737 is a Bcl-2 and Bcl-xL specific BH3 mimetic, it does not target Mcl-1 to have any additional effect in cyclin E-depleted Reh ABT-R cells. ABT-737 treatment in ABT-R cells triggers activation of MAPK/ERK pathways that have been shown to phosphorylate Mcl-1 at Thr163 residue [9] and it plays additional role in Mcl-1 stability [17]. Nevertheless, since Mcl-1 levels were down-regulated following cyclin E depletion that resulted in caspase-3 cleavage, it suggests cyclin E/Cdk2-mediated regulation of Mcl-1 in ABT-R cells.

Since therapeutic strategies that utilize Mcl-1-specific inhibitors have so far been ineffective and that Mcl-1 deletion may have serious side effects [26, 34, 35], there is a need for novel ways to target Mcl-1 regulation to overcome its role in chemoresistance [1]. Aberrant activation of CDKs leads to cell-cycle dysregulation, which is a hallmark of all human cancer. CDK inhibition for targeting cancer has shown therapeutic potential and subsequently many pan-CDK inhibitors have been considered for clinical development. However, their non-specific activity has caused undesirable effects and hindered their further development [36]. Dinaciclib, a potent selective multi-CDK inhibitor (Cdk1, Cdk2, Cdk5, and Cdk9) is under clinical development [36-38]. As compared to previous CDK inhibitors such as flavopiridol, it has shown reduced toxicity and enhanced clinical efficiency in patients with CLL, multiple myeloma, and solid tumors [36, 39, 40]. Higher concentrations of dinaciclib were shown to down-regulate Mcl-1 levels by targeting its transcriptional control by Cdk9 [41]. Our results show that dinaciclib at a concentration of 10 nM, when combined with ABT-199 or ABT-737, shows potent synergistic cell death in leukemic cell lines and primary CLL patient samples. Low concentration of dinaciclib would be desirable and achievable in certain clinical settings. Dinaciclib disrupted the Mcl-1/Bim interaction in Reh ABT-R cells with no changes in protein and mRNA levels. This indicates that Mcl-1, which was phosphorylated at Ser64 and Thr163 in ABT-R cells [17], is inhibited by dinaciclib treatment, resulting in Bim release and subsequent apoptosis.

Taken together, our findings provide a mechanism for cyclin E/Cdk2-mediated phosphorylation of Mcl-1 as a determinant of enhanced Mcl-1 stability and function. Cdk inhibitors can target the increased Mcl-1 levels that are critical for mediating chemoresistance. Our data support a rational for using dinaciclib in combination with venetoclax in clinical trials.

## MATERIALS AND METHODS

### Cell lines, plasmids, and reagents

PC3, H1299, HEK293T, and Reh cell lines were purchased from the American Type Culture Collection (ATCC, Vanassas, MA). DOHH2 and SU-DHL-16 were as described [18]. WT and cyclin E−/− MEFs were from Dr. Piotr Sicinski (Harvard Medical School) [19]. PC3, DOHH2, SU-DHL-16, and Reh were cultured in RPMI-1640; H1299 MEF and HEK293T in DMEM. All media were supplemented with 10% fetal bovine serum (FBS; Gibco BRL), penicillin-streptomycin and antibiotic-antimycotic (Gibco, Life Technologies, Gaithersburg, MD). Reh ABT-R cell lines were generated and cultured as described [17]. Myc-Mcl-1 was a gift from Dr. Wenyi Wei (Harvard Medical School). HA-cyclin E was used as described [42]. The Mcl-1 mutants were generated using QuikChange II XL Site-Directed Mutagenesis Kit (Agilent Technologies). siMcl-1, siControl (Dharmacon-GE); siCyclin A, siControl (Santa Cruz Biotechnology, Santa Cruz, CA) were transfected with Lipofectamine 2000 (Life Technologies, Carlsbad, CA) according to manufacture's instructions. Reh ABT-R cells were transfected with siControl or siMcl-1 using an Amaxa Nucleofector Kit R (Lonza, Walkersville, MD) (program number I-009) according to the manufacture's protocol. ABT-737 and ABT-199 were obtained from AbbVie (Chicago, IL, USA), dinaciclib from Selleck Chemicals (Houston, TX, USA), and cycloheximide (20 μg/ml) from Sigma-Aldrich (St. Louis, MO).

### Patient samples

Peripheral blood samples from CLL patients were obtained with the patients' informed consent according to protocols approved by the Cleveland Clinic Institutional Review Board according to the Declaration of Helsinki. Lymphocytes were purified by Ficoll-Paque PLUS (GE Healthcare Bio-Sciences, Pittsburgh) gradient centrifugation and used immediately [25].

### Immunoblotting and immunoprecipitation

Cell pellets were lysed and protein lysates were prepared as described [17]. Proteins were resolved on SDS-PAGE followed by transferring to nitrocellulose membranes and incubated with primary antibody. For ubiquitination experiments, MG132 and N-ethylmaleimide (Sigma-Aldrich) were added to lysis buffer with dithiothreitol (Sigma-Aldrich) to prepare lysates. Immunoprecipitation was performed as described [17]. Briefly, cells were lysed with CHAPS (Sigma) buffer and equal amount of protein were incubated with primary antibody overnight at 4°C. Protein A agarose beads (Rockland Immunochemicals, Gilbertsville, PA) were added to all samples, followed by 1 h of incubation at 4°C. After washing 3 times with CHAPS, the beads were eluted with loading buffer supplemented with 2-mercaptoethanol (Sigma-Aldrich) and immunoblotting was performed. Primary antibodies were for Mcl-1, Bim (BD-Biosciences), Mcl-1 (Rockland Immunochemicals, Gilbertsville, PA) Bcl-2, Bcl-xL, cyclin E (HE12), cyclin E (HE111), cyclin E (M-20), cyclin B1, cyclin A (H432), Cdk2 (M2) (Santa Cruz Biotechnology, Santa Cruz, CA), cleaved caspase-3 (Cell Signaling), and β-actin (Sigma-Aldrich). The secondary anti-mouse and -rabbit antibodies were purchased from Thermo-Fisher Scientific (Waltham, MA). Protein levels were quantified by ImageJ (NIH, Bethesda, MD). The relative intensity of each lane with respect to control at 0 h was calculated after normalizing it to the relative intensity of β-actin. The Myc-Mcl-1 and HA-cyclin E constructs expressed in MEF cells were detected using ant-Mcl-1 and cyclin E antibodies that did not recognize endogenous proteins.

### RNA isolation and real-time quantitative-PCR

RNA was isolated by the Trizol method (Life Technologies) and a quantitative real-time, reverse transcriptase PCR (qRT-PCR) kit (Life Technologies) was used to analyze mRNA levels in human cells using primers for *Mcl-1*, and normalized to *β-actin*, as described [17]. Primers for *Mcl-1 in* MEFs were forward 5′-AGGCGGCATCAGAAATGTG-3′ reverse 5′-CAGCCCCTACTCCAGCAACA-3′ and *β-Actin,* forward 5′-CGATGCCCTGAGGCTCTTT-3′ reverse 5′-TGGATGCCACAGGATTCCA-3′.

### Cell viability and cell cycle analysis

Cell viability was determined as described [17] using fluorescein-conjugated Annexin V-PI (BD Biosciences) on a BD FACS Calibur flow cytometer. In order to measure cell viability, 3-(4,5-dimethylthiazol-2-yl)-5-(3-carboxymethoxyphenyl)-2-(4-sulfophenyl)-2H tetrazolium inner salt (MTS) assay (Promega, Madison, WI, USA) was used and percentage reduction in metabolic activity was calculated as described [43]. For cell cycle analysis, cells were fixed with 70% ethanol and incubated on ice for 15 min. The cells were then stained with solution containing propidium iodide (PI) (50 μg/ml), RNase A (0.1 mg/ml), Triton X (0.05%) for 40 min at 37°C. Following incubation, 500 μl of PBS was added to each sample and cells were analyzed on a BD FACS Calibur flow cytometer (Beckton Dickinson, Mansfield, MA). The data for cell viability and cell cycle were processed using the CellQuest Version 5.2.1 software. The results were normalized to survival of vehicle control cells treated with ethanol or DMSO (Sigma-Aldrich).

### Synergy analysis

Combination Index values were calculated as indicated previously [18, 43] using the Chou - Talay method and Calcusyn software (Biosoft, Cambridge, UK). CI values <1.0 indicate synergism.

### Statistical analysis

Statistical comparisons between groups were conducted using a 2-tailed student's *t*-test and one-way ANOVA. Protein half-life was analyzed using the one-phase exponential decay model in Prism (Version 4.0c, GraphPad Software Inc). Standard deviation (SD) was calculated from experiments conducted in triplicate and is indicated by error bars on the figures. All experiments were repeated three times, independently.

## SUPPLEMENTARY MATERIAL FIGURES



## References

[1] Ertel F, Nguyen M, Roulston A, Shore GC (2013). Programming cancer cells for high expression levels of Mcl1. EMBO Rep.

[2] Siu KT, Rosner MR, Minella AC (2012). An integrated view of cyclin E function and regulation. Cell Cycle.

[3] Mazumder S, Gong B, Almasan A (2000). Cyclin E induction by genotoxic stress leads to apoptosis of hematopoietic cells. Oncogene.

[4] Mazumder S, DuPree EL, Almasan A (2004). A dual role of cyclin E in cell proliferation and apoptosis may provide a target for cancer therapy. Curr Cancer Drug Targets.

[5] Hwang HC, Clurman BE (2005). Cyclin E in normal and neoplastic cell cycles. Oncogene.

[6] Harley ME, Allan LA, Sanderson HS, Clarke PR (2010). Phosphorylation of Mcl-1 by CDK1-cyclin B1 initiates its Cdc20-dependent destruction during mitotic arrest. EMBO J.

[7] Kobayashi S, Lee SH, Meng XW, Mott JL, Bronk SF, Werneburg NW, Craig RW, Kaufmann SH, Gores GJ (2007). Serine 64 phosphorylation enhances the antiapoptotic function of Mcl-1. J Biol Chem.

[8] Thomas LW, Lam C, Edwards SW (2010). Mcl-1; the molecular regulation of protein function. FEBS Lett.

[9] Morel C, Carlson SM, White FM, Davis RJ (2009). Mcl-1 integrates the opposing actions of signaling pathways that mediate survival and apoptosis. Mol Cell Biol.

[10] Nifoussi SK, Vrana JA, Domina AM, De Biasio A, Gui J, Gregory MA, Hann SR, Craig RW (2012). Thr 163 phosphorylation causes Mcl-1 stabilization when degradation is independent of the adjacent GSK3-targeted phosphodegron, promoting drug resistance in cancer. PLoS One.

[11] Maurer U, Charvet C, Wagman AS, Dejardin E, Green DR (2006). Glycogen synthase kinase-3 regulates mitochondrial outer membrane permeabilization and apoptosis by destabilization of MCL-1. Mol Cell.

[12] Opferman JT (2006). Unraveling MCL-1 degradation. Cell Death Differ.

[13] Wertz IE, Kusam S, Lam C, Okamoto T, Sandoval W, Anderson DJ, Helgason E, Ernst JA, Eby M, Liu J, Belmont LD, Kaminker JS, O'Rourke KM, Pujara K, Kohli PB, Johnson AR (2011). Sensitivity to antitubulin chemotherapeutics is regulated by MCL1 and FBW7. Nature.

[14] Scaltriti M, Eichhorn PJ, Cortes J, Prudkin L, Aura C, Jimenez J, Chandarlapaty S, Serra V, Prat A, Ibrahim YH, Guzman M, Gili M, Rodriguez O, Rodriguez S, Perez J, Green SR (2011). Cyclin E amplification/overexpression is a mechanism of trastuzumab resistance in HER2+ breast cancer patients. Proc Natl Acad Sci U S A.

[15] Juin P, Geneste O, Gautier F, Depil S, Campone M (2013). Decoding and unlocking the BCL-2 dependency of cancer cells. Nat Rev Cancer.

[16] Davids MS, Seymour JF, Gerecitano JF, Kahl BS, Pagel JM, Wierda WG, Anderson MA, Rudersdorf NK, Gressick LA, Montalvo NP, Yang JN, Busman TA, Dunbar M, Cerri E, Enschede SH, Humerickhouse RA (2013). The Single-Agent Bcl-2 Inhibitor ABT-199 (GDC-0199) In patients with relapsed/refractory (R/R) non-hodgkin lymphoma (NHL): responses observed in all mantle cell lymphoma (MCL) patients. Blood.

[17] Mazumder S, Choudhary GS, Al-Harbi S, Almasan A (2012). Mcl-1 Phosphorylation defines ABT-737 resistance that can be overcome by increased NOXA expression in leukemic B cells. Cancer Res.

[18] Choudhary GS, Al-Harbi S, Mazumder S, Hill BT, Smith MR, Bodo J, Hsi ED, Almasan A (2015). MCL-1 and BCL-xL-dependent resistance to the BCL-2 inhibitor ABT-199 can be overcome by preventing PI3K/AKT/mTOR activation in lymphoid malignancies. Cell Death Dis.

[19] Geng Y, Yu Q, Sicinska E, Das M, Schneider JE, Bhattacharya S, Rideout WM, Bronson RT, Gardner H, Sicinski P (2003). Cyclin E ablation in the mouse. Cell.

[20] Stevenson-Lindert LM, Fowler P, Lew J (2003). Substrate specificity of CDK2-cyclin A. What is optimal?. J Biol Chem.

[21] van Delft MF, Wei AH, Mason KD, Vandenberg CJ, Chen L, Czabotar PE, Willis SN, Scott CL, Day CL, Cory S, Adams JM, Roberts AW, Huang DC (2006). The BH3 mimetic ABT-737 targets selective Bcl-2 proteins and efficiently induces apoptosis via Bak/Bax if Mcl-1 is neutralized. Cancer Cell.

[22] Souers AJ, Leverson JD, Boghaert ER, Ackler SL, Catron ND, Chen J, Dayton BD, Ding H, Enschede SH, Fairbrother WJ, Huang DC, Hymowitz SG, Jin S, Khaw SL, Kovar PJ, Lam LT (2013). ABT-199, a potent and selective BCL-2 inhibitor, achieves antitumor activity while sparing platelets. Nat Med.

[23] Ding Q, He X, Hsu JM, Xia W, Chen CT, Li LY, Lee DF, Liu JC, Zhong Q, Wang X, Hung MC (2007). Degradation of Mcl-1 by beta-TrCP mediates glycogen synthase kinase 3-induced tumor suppression and chemosensitization. Mol Cell Biol.

[24] Ding Q, Huo L, Yang JY, Xia W, Wei Y, Liao Y, Chang CJ, Yang Y, Lai CC, Lee DF, Yen CJ, Chen YJ, Hsu JM, Kuo HP, Lin CY, Tsai FJ (2008). Down-regulation of myeloid cell leukemia-1 through inhibiting Erk/Pin 1 pathway by sorafenib facilitates chemosensitization in breast cancer. Cancer Res.

[25] Al-Harbi S, Hill BT, Mazumder S, Singh K, Devecchio J, Choudhary G, Rybicki LA, Kalaycio M, Maciejewski JP, Houghton JA, Almasan A (2011). An antiapoptotic BCL-2 family expression index predicts the response of chronic lymphocytic leukemia to ABT-737. Blood.

[26] Thomas RL, Roberts DJ, Kubli DA, Lee Y, Quinsay MN, Owens JB, Fischer KM, Sussman MA, Miyamoto S, Gustafsson AB (2013). Loss of MCL-1 leads to impaired autophagy and rapid development of heart failure. Genes Dev.

[27] Inoshita S, Takeda K, Hatai T, Terada Y, Sano M, Hata J, Umezawa A, Ichijo H (2002). Phosphorylation and inactivation of myeloid cell leukemia 1 by JNK in response to oxidative stress. J Biol Chem.

[28] Inuzuka H, Shaik S, Onoyama I, Gao D, Tseng A, Maser RS, Zhai B, Wan L, Gutierrez A, Lau AW, Xiao Y, Christie AL, Aster J, Settleman J, Gygi SP, Kung AL (2011). SCF(FBW7) regulates cellular apoptosis by targeting MCL1 for ubiquitylation and destruction. Nature.

[29] Inuzuka H, Fukushima H, Shaik S, Liu P, Lau AW, Wei W (2011). Mcl-1 ubiquitination and destruction. Oncotarget.

[30] Welcker M, Clurman BE (2008). FBW7 ubiquitin ligase: a tumour suppressor at the crossroads of cell division, growth and differentiation. Nat Rev Cancer.

[31] Wang Z, Inuzuka H, Fukushima H, Wan L, Gao D, Shaik S, Sarkar FH, Wei W (2012). Emerging roles of the FBW7 tumour suppressor in stem cell differentiation. EMBO Rep.

[32] Konopleva M, Milella M, Ruvolo P, Watts JC, Ricciardi MR, Korchin B, McQueen T, Bornmann W, Tsao T, Bergamo P, Mak DH, Chen W, McCubrey J, Tafuri A, Andreeff M (2012). MEK inhibition enhances ABT-737-induced leukemia cell apoptosis via prevention of ERK-activated MCL-1 induction and modulation of MCL-1/BIM complex. Leukemia.

[33] Rahmani M, Aust MM, Attkisson E, Williams DC, Ferreira-Gonzalez A, Grant S (2013). Dual inhibition of Bcl-2 and Bcl-xL strikingly enhances PI3K inhibition-induced apoptosis in human myeloid leukemia cells through a GSK3- and Bim-dependent mechanism. Cancer Res.

[34] Perciavalle RM, Opferman JT (2013). Delving deeper: MCL-1's contributions to normal and cancer biology. Trends Cell Biol.

[35] Wang X, Bathina M, Lynch J, Koss B, Calabrese C, Frase S, Schuetz JD, Rehg JE, Opferman JT (2013). Deletion of MCL-1 causes lethal cardiac failure and mitochondrial dysfunction. Genes Dev.

[36] Parry D, Guzi T, Shanahan F, Davis N, Prabhavalkar D, Wiswell D, Seghezzi W, Paruch K, Dwyer MP, Doll R, Nomeir A, Windsor W, Fischmann T, Wang Y, Oft M, Chen T (2010). Dinaciclib (SCH 727965), a novel and potent cyclin-dependent kinase inhibitor. Mol Cancer Ther.

[37] Johnson AJ, Yeh YY, Smith LL, Wagner AJ, Hessler J, Gupta S, Flynn J, Jones J, Zhang X, Bannerji R, Grever MR, Byrd JC (2012). The novel cyclin-dependent kinase inhibitor dinaciclib (SCH727965) promotes apoptosis and abrogates microenvironmental cytokine protection in chronic lymphocytic leukemia cells. Leukemia.

[38] Flynn J, Jones J, Johnson AJ, Andritsos L, Maddocks K, Jaglowski S, Hessler J, Grever MR, Im E, Zhou H, Zhu Y, Zhang D, Small K, Bannerji R, Byrd JC (2015). Dinaciclib is a novel cyclin-dependent kinase inhibitor with significant clinical activity in relapsed and refractory chronic lymphocytic leukemia. Leukemia.

[39] Kumar SK, LaPlant B, Chng WJ, Zonder J, Callander N, Fonseca R, Fruth B, Roy V, Erlichman C, Stewart AK (2015). Dinaciclib, a novel CDK inhibitor, demonstrates encouraging single-agent activity in patients with relapsed multiple myeloma. Blood.

[40] Billard C (2014). Apoptosis inducers in chronic lymphocytic leukemia. Oncotarget.

[41] Booher RN, Hatch H, Dolinski BM, Nguyen T, Harmonay L, Al-Assaad AS, Ayers M, Nebozhyn M, Loboda A, Hirsch HA, Zhang T, Shi B, Merkel CE, Angagaw MH, Wang Y, Long BJ (2014). MCL1 and BCL-xL levels in solid tumors are predictive of dinaciclib-induced apoptosis. PLoS One.

[42] Plesca D, Mazumder S, Gama V, Matsuyama S, Almasan A (2008). A C-terminal fragment of Cyclin E, generated by caspase-mediated cleavage, is degraded in the absence of a recognizable phosphodegron. J Biol Chem.

[43] Sharma A, Singh K, Mazumder S, Hill BT, Kalaycio M, Almasan A (2013). BECN1 and BIM interactions with MCL-1 determine fludarabine resistance in leukemic B cells. Cell Death Dis.

