# Correction: Cyclin E/Cdk2-dependent phosphorylation of Mcl-1 determines its stability and cellular sensitivity to BH3 mimetics

**DOI:** 10.18632/oncotarget.28593

**Published:** 2024-07-12

**Authors:** Gaurav S. Choudhary, Trinh T. Tat, Saurav Misra, Brian T. Hill, Mitchell R. Smith, Alexandru Almasan, Suparna Mazumder

**Affiliations:** ^1^Department of Cancer Biology, Lerner Research Institute, Cleveland Clinic, Cleveland, OH, USA; ^2^Department of Molecular Cardiology, Lerner Research Institute, Cleveland Clinic, Cleveland, OH, USA; ^3^Department of Immunology, Lerner Research Institute, Taussig Cancer Institute, Cleveland Clinic, Cleveland, OH, USA; ^4^Department of Hematology and Oncology, Taussig Cancer Institute, Cleveland Clinic, Cleveland, OH, USA; ^5^Department of Molecular Medicine, Cleveland Clinic Lerner College of Medicine of Case Western Reserve University, Cleveland, OH, USA; ^6^Department of Pathology, Case Western Reserve University School of Medicine, Cleveland, OH, USA; ^7^Department of Biochemistry, Case Western Reserve University School of Medicine, Cleveland, OH, USA

**This article has been corrected:** In Figure 4H, the first two panels in the β-actin row are accidental duplicate images. As a result, these changes alter the ratio of Mcl1/b-actin for this panel, which is presented on the graph (panel I). The corrected Figure 4 with new images for panel H and panel I, obtained using the original data, is shown below. The authors declare that these corrections do not change the results or conclusions of this paper.

Original article: Oncotarget. 2015; 6:16912–16925. 16912-16925. https://doi.org/10.18632/oncotarget.4857

**Figure 4:**
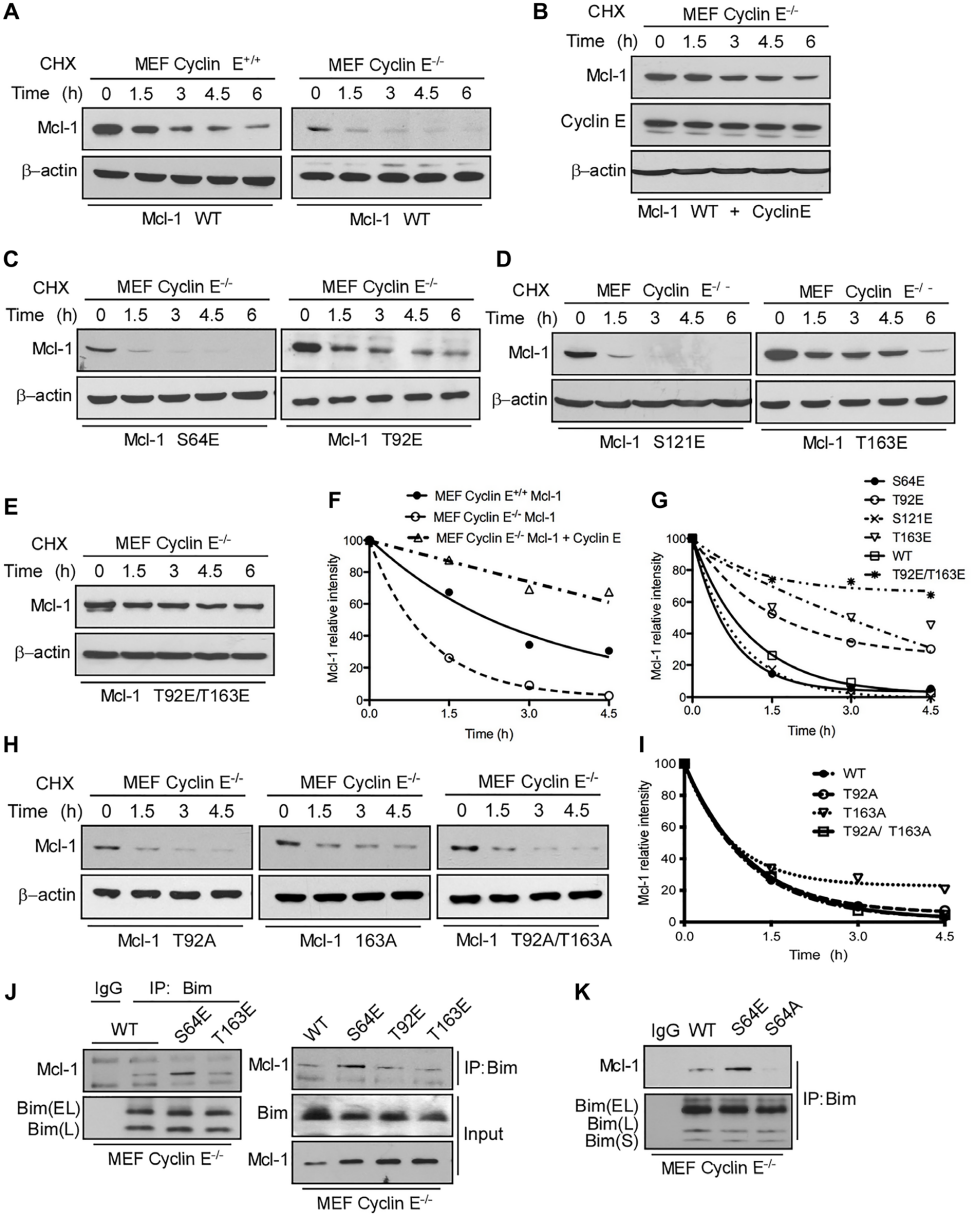
Mcl-1 stability and Bim sequestration is dependent on cyclin E/Cdk2. Mcl-1 protein half-life was determined by expressing WT Myc-Mcl-1 (**A**). individually in cyclin E^+/+^ and cyclin E^−/−^ MEFs (**B**). together with HA-cyclin E in cyclin E^−/−^ MEFs and then treating with cycloheximide for the indicated time, followed by immunoblotting. Immunoblot analysis of cyclin E^−/−^ MEFs transfected with (**C**). S64E, T92E, (**D**). S121E, T163E (**E**). T92E/T163E (**H**). T92A, T163A and T92A/T163A Mcl-1 mutants and treated with cycloheximide for the indicated time. Data in (**F**)., (**G**)., (**I**). were quantified by ImageJ. Cyclin E^−/−^ MEFs were transfected with (**J**). Myc-Mcl-1 WT, S64E, T92E and T163E (**K**). Myc-Mcl-1 WT, S64E and S64A. After 24 h, Bim was immunoprecipitated and its association with Mcl-1 was analyzed by immunoblotting. β-actin was used as loading control. These data are representative of three independent experiments.

