# Supplementary material for: Cyclin E/Cdk2-dependent phosphorylation of Mcl-1 determines its stability and cellular sensitivity to BH3 mimetics
Source: Oncotarget. 2015 Jul 15;6(19):16912–25. doi: 10.18632/oncotarget.4857 (PMC4627281; doi:10.18632/oncotarget.4857)
Supplement: Supplementary file 1 [file oncotarget-06-16912-s001.pdf]

Cyclin E/Cdk2-dependent phosphorylation of Mcl-1 determines its stability and cellular sensitivity to BH3 mimetics

Supplementary Material

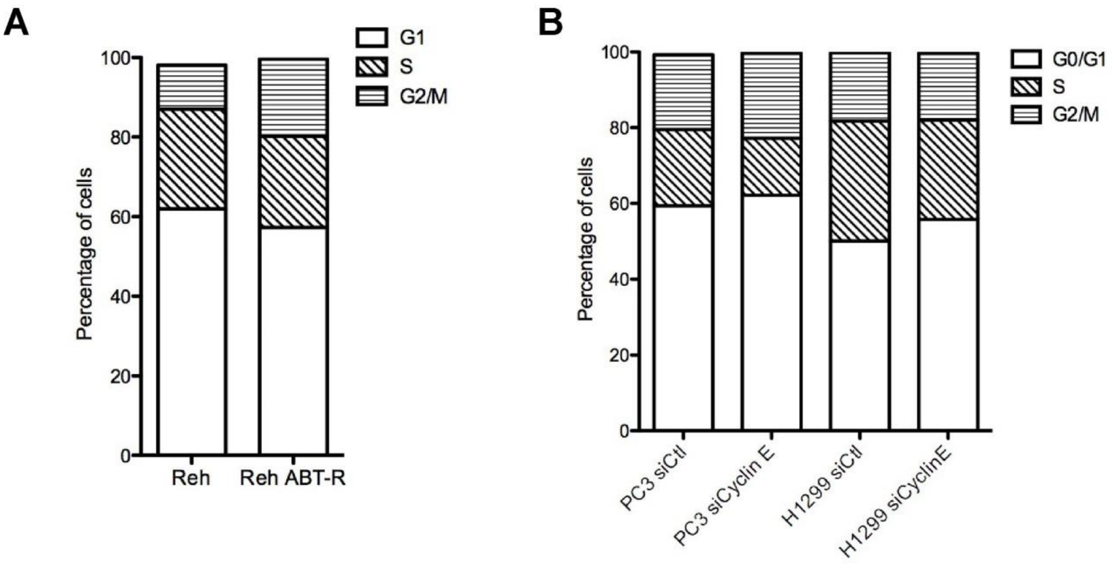

Figure S1: Cell-cycle analysis. Cell cycle distribution of (**A**) Reh and Reh ABT-R (**B**) PC3 and H1299 cells transfected with siCyclin E and siControl, were analyzed by flow cytometry. The data are representative of three independent experiments.

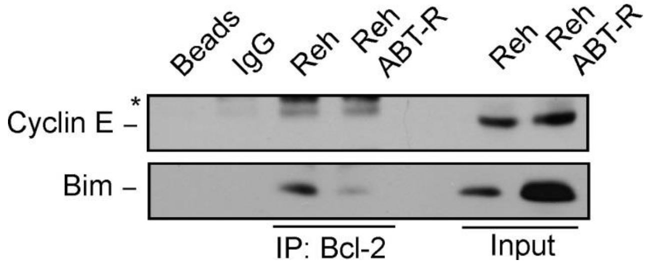

Figure S2: Bcl-2 is not associated with the Cyclin E/CDK2 complex. Bcl-2 was immunoprecipitated from Reh parental and ABT-R-derivative cell lysates and its association with Cyclin E and Bim was determined by immunoblotting with Cyclin E and Bim specific antibodies. *non-specific band.

A

61	AGA**S**PPS	*67*	Mcl-1
89	VTA**T**PAR	*95*	Mcl-1
160	LPS**T**PPP	*166*	Mcl-1
564	LSD**S**PLF	*570*	Rb
719	QVH**T**PKT	*725*	Mcm3
211	LYR**S**PSM	*217*	Cdc25C
198	DAS**S**PRS	*204*	MyoD
184	VEQ**T**PKK	*190*	p27Kip1
196	IRD**T**PAK	*202*	Nucleophosmin
248	TSV**T**PQK	*254*	E2F-5
85	TPV**T**PKK	*91*	Hbo1/KAT7
24	VAS**S**PSK	*30*	MARCKS
552	VLT**T**PSK	*558*	HIRA/TUPLE1
1494	ERS**S**PSK	*1500*	Brca1
597	PFK**T**PIK	*603*	FoxM1/FKHL16
608	ISS**T**PSK	*614*	FoxM1/FKHL16
635	LDF**S**PVQ	*641*	FoxM1/FKHL16
772	ILS**S**PTK	*778*	NPAT/p220
776	PTK**S**PTK	*782*	NPAT/p220
1097	NLD**S**PNV	*1103*	NPAT/p220
1267	VPR**T**PGS	*1273*	NPAT/p220
1347	TSA**T**PLK	*1353*	NPAT/p220
	HHA**S**PRK		Peptide Library

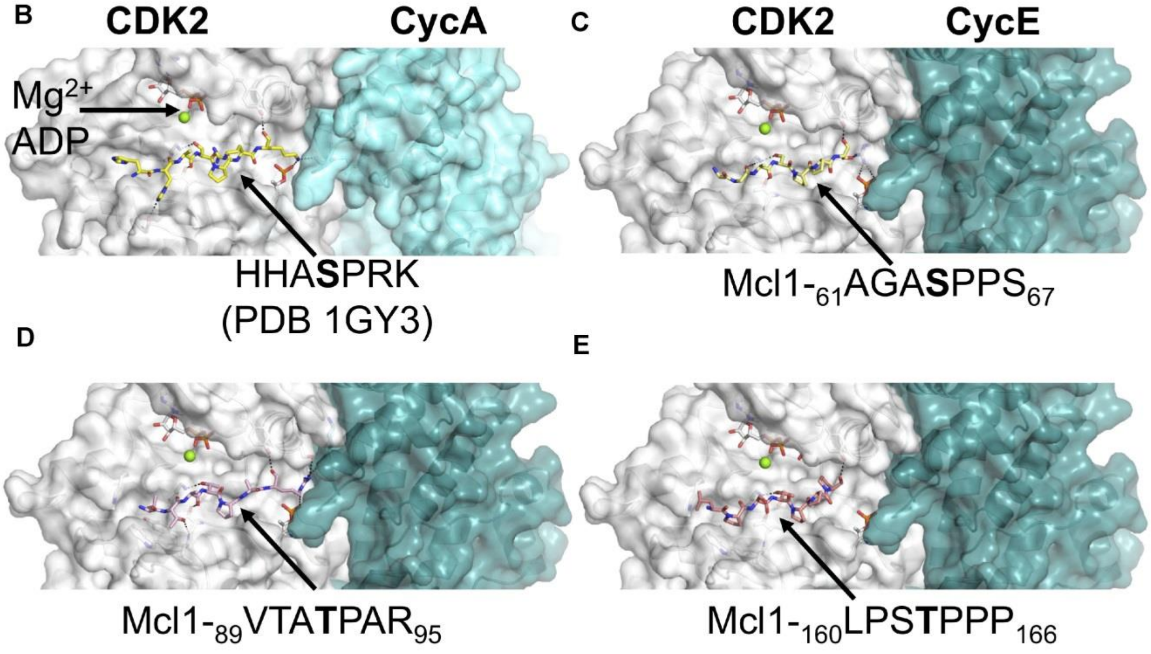

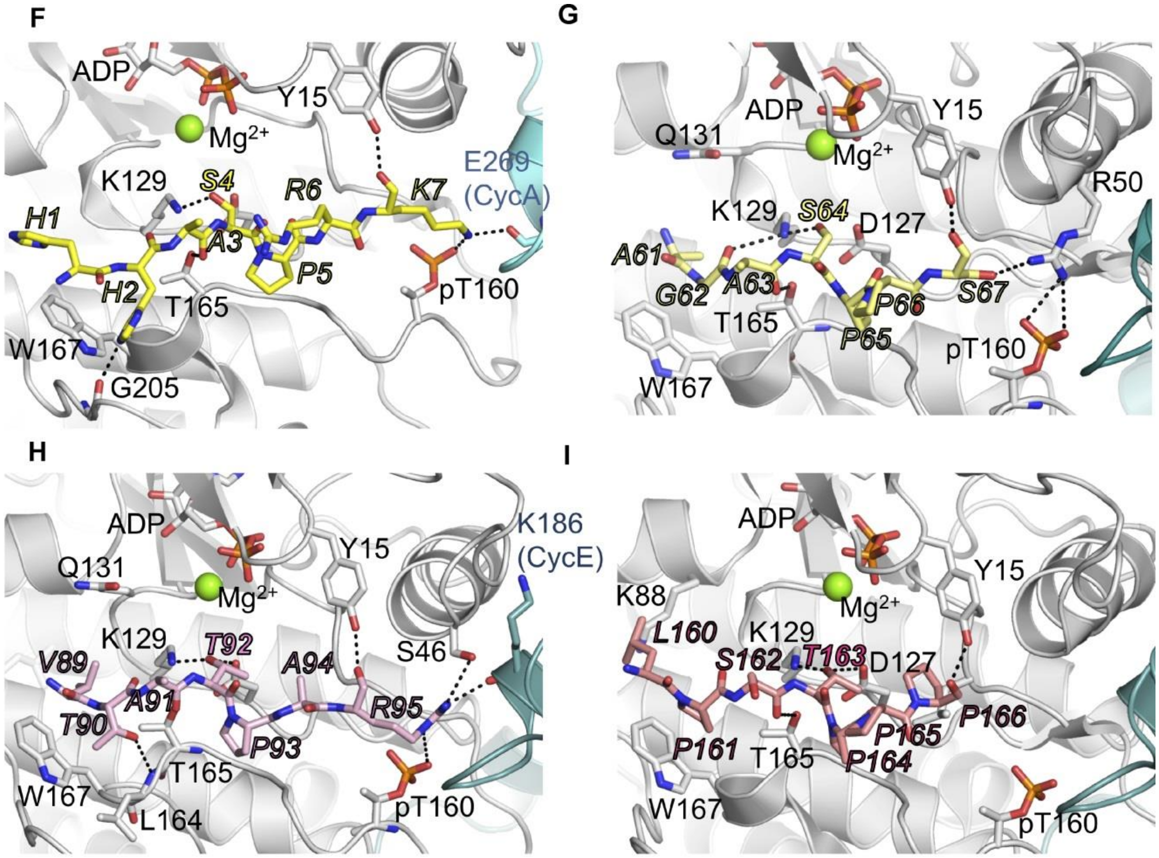

Figure S3: Identification of Mcl-1 phosphorylation sites by Cyclin E/Cdk2 and their comparison with other known Cdk2 substrates. (**A**) Sequence alignment of the optimum amino-acid sequence predicted for Cdk2 with Mcl-1 and known Cdk2 phosphorylation substrate motifs. (**B**) Details of binding of an optimum substrate peptide (yellow) bound to the active site of Cdk2 in the cyclin A/Cdk2 complex (PDB 1GY3; [1]. The peptide, key Cdk2 and cyclin A residues, and ADP are shown in stick representation, while the bound magnesium counterion is shown as a green sphere. T160 is phosphorylated (“pT160”). Dotted lines designate likely salt bridges or hydrogen bonds. Details of models of sequences representing the (**C**) Ser64 (**D**) Thr92 and (**E**) Thr163 phosphorylation sites of Mcl-1 (light yellow, light pink or medium pink) bound to the active site of Cdk2 in the cyclin E/Cdk2 complex. Mcl-1 residues are labeled in italics. (**F**) Surface representation of Cdk2/Cyclin A crystal structure bound to an

“optimum” Cdk2 substrate peptide identified from a peptide library [2], from PDB 1GY3 [1]. (**G-I**) This structure was combined with a structure of Cdk2-Cyclin E1 (PDB 1W98; [3] as a template for modeling of Cdk2/Cyclin E bound to the target sequences on Mcl-1-Ser64, Thr92, and Thr163. The models were generated using FlexPepDock [4]. Surface representations of the resulting Cdk2/Cyclin E complexes with the corresponding target sequences are shown. The top-scoring output from each FlexPepDock run is shown.

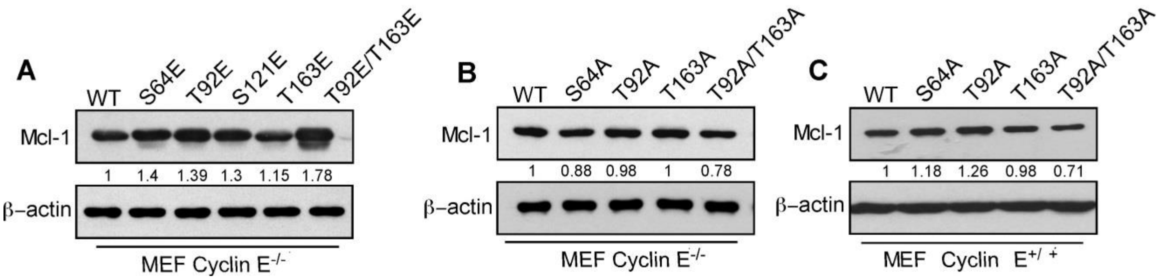

Figure S4: Expression of Mcl-1 mutants in WT and cyclin $E^{-/-}$ MEFs. Immunoblot analysis for Mcl-1 after expressing (**A**) Myc-Mcl-1 WT, S64E, T92E, S121E, T163E, and T92E/T163E constructs in WT MEFs and Myc-Mcl-1 WT, S64A, T92A, T163A, and T92A/T163A constructs in (**B**) cyclin $E^{-/-}$, and (**C**) WT MEFs. β-actin was used as a loading control. Data in **A-C** were quantified by ImageJ. These data are representative of three independent experiments.

References:

1. Cook A, Lowe ED, Chrysina ED, Skamnaki VT, Oikonomakos NG and Johnson LN. Structural studies on phospho-CDK2/cyclin A bound to nitrate, a transition state analogue: implications for the protein kinase mechanism. Biochemistry. 2002; 41:7301-7311.
2. Songyang Z, Blechner S, Hoagland N, Hoekstra MF, Piwnica-Worms H and Cantley LC. Use of an oriented peptide library to determine the optimal substrates of protein kinases. Curr Biol. 1994; 4:973-982.
3. Honda R, Lowe ED, Dubinina E, Skamnaki V, Cook A, Brown NR and Johnson LN. The structure of cyclin E1/CDK2: implications for CDK2 activation and CDK2-independent roles. EMBO J. 2005; 24:452-463.
4. Raveh B, London N and Scheler-Furman O. Sub-angstrom modeling of complexes between flexible peptides and globular proteins. Proteins. 2010; 78:2029-2040.
